# The effects of ultra-selective beta1-antagonism on the metabolic and cytokine profile in septic shock patients receiving noradrenaline: a sub-investigation from the STRESS-L Randomised Study

**DOI:** 10.1186/s40635-024-00708-6

**Published:** 2025-01-22

**Authors:** Jarrod L. Thomas, Kirsty C. McGee, Anower Hossain, Gavin D. Perkins, Anthony C. Gordon, Duncan Young, Danny McAuley, Mervyn Singer, Ranjit Lall, Tina Kramaric, Janet M. Lord, Tony Whitehouse, Luis A. J. Mur, for the STRESS-L. collaborators, Mervyn Singer, Tonny Veenith, Jaimin Patel, Nick Murphy, Mansoor Bangash, Tomasz Torlinski, Nicholas Talbot, Catherine Snelson, Dhruv Parekh, Amisha Desai, Mary Kotada, Yin May Chin, Sophie Holden, Aoife Neal, Maximina Ventura, Martin Pope, Samantha Harkett, Christopher McGhee, Emma Fellows, Amy Bamford, Ronald Carrera, Karen Ellis, Elaine Spruce, Liesl Despy, Stephanie Porter, Colin Bergin, Stephanie Goundry, Hazel Smith, Tracy Mason, Natalie Dooley, Amy Clark, Joyce Yeung, Jo Gresty, Teresa Melody, Ellie Reeves, Rachel Smith, Julia Sampson, Chris Nutt, James Mcnamee, Danny Mcauley, Andren Boyle, Loren McGinley, Chris Wright, Kathryn Ward, Lauren Macartney, Justine Mccann, Brian Wells, Janette Mills, Leona Bannon, Aisling O’Neill, Stephanie Finn, Anthony Gordon, David Antcliffe, Stephen Brett, Dorota Banach, Leilani Cabreros, Laura Curran, Sonia Sousa Arias, Roceld Rojo, Ziortza Fernandez de Pinedo Artaraz, Phoebe Coghlan, Maie Templeton, Ahmed ElHaddad, Zohonon Sabine Loko, Gareth Barker, Niall MacCallum, David Brealey, Kristian Warnes, Nina Bason, Dorota Filipowicz, Georgia Bercades, Ingrid Hass, Jung Ryu, Deborah Smyth, Dorothy Ilano, Dan Harvey, Louise Conner, Lucy Ryan, Cecilia Peters, Megan Meredith, Megan Mcaulay, Zoe Whitman, Amy Clark, Lucy Morris, Julia Sampson, Claudia Woodford, Sally Hodgkinson, Sonya Finucane, Luigi Camporota, Manu Shankar-Hari, Marlies Ostermann, Aneta Bociek, Rosario Lim, Neus GrauNovellas, Natalie Palmer, Angela Cape, Andrea Kelly, Gill Arbane, Sarah Campos, Michael Singh, Jinny Yoo, Richard Innes, Fiona Dempsey, Patricia Doble, Rebecca Purnell, Moira Tait, Jo Hutter, Corinne Pawley, Joy Rowe, Catherine Wane, Richard Burgess, Ashly Thomas, Pulak Paul, Brenda White, Wayne Lovegrove, Mandy Gill, Lynne Wade, Tracy Brear, Vishal Dhokia, Debbie Jackson, Sarah Shelton, Jill Kirk, Andrew Boulton, Alistair Roy, Anthony Rostron, Zeynep Elcioglu, Lindsey Woods, Sarah Cornell, Rebecca Betts, Jill Holden, Stephen Laybourne, Kimberley Rogerson, Jeremy Bewley, Rebekah Johnson, Anna Chillingworth, Bethany Gumbrill, Hilary Galvin, Kim Wright, Georgia Efford, Kathleen Corcoran, Libby Cole, Katie Sweet, Denise Webster, Lisa Grimmer, Gemma Paris, Chloe Searles, Zoe Garland, Charly Gibson, Melanie Hutchings, Fiona Walters, Sinead Kelly, Lorraine Scaife, Kelly Littlewood, James Leavy, Shirley Todd, Elizabeth Gordon, Sadie Heddon, Victor Mariano, Karin Gupwell, Rebecca Appelboam, Samantha Keenan, Cassie Brady, Ian Mew, Duncan Chambler, Sarah Williams, Pauline Ashcroft, Patricia Williams, James Rees, Sophie Wiseman, Sarah Horton, Mark Shortland, Stephanie Dukes, Andy Ball, Michael Spivey, Jennie Stephens, Sarah Bean, Karen Burt, Rachel Chapman, Carol Richards, Lorraine Moore, Linda Allsop, Amber Wynn, Louise Latty, Sam Waddy, Kayleigh Spencer, Bethan Stowe, Georgina Cox, Helen McMillan, Liana Lankester, Colin Wells, Rosalyn Squire, Mike Marner, Robert Jackson, Nikitas Nikitas, Stuart Dickson, Henrik Reschreiter, James Bromilow, James Keegan, Chris Loew, Ken Power, Spike Briggs, Julie Camsooksai, Sarah Patch, Sarah Jenkins, Sharon Power, Elizabeth Woodward, David Pogson, Zoe Daly, Steve Rose, Aimi Collins, Amy Phelps, Helen Claridge, Christine Minnis, Sarah Inglis, Lutece Brimfield, Charlotte Wong, Ingeborg Welters, Karen Williams, Victoria Waugh, Julie Patrick-Heselton, Emily Johnson, David Shaw, Brian Johnston, Maryam Crews, Alicia Waite, Vinoth Sankar, Jonathan Walker, Peter Turton, Richard Wenstone, Jaime Fernandez Roman, Alison Hall, Maria Lopez Martinez, David Oliver Hamilton, David Coey, Philip Shelley, Martin Goulding, Karen Kavanagh, Ann Marron, Hannah Allsop, Phil Hopkins, John Smith, Harriet Noble, Evita Pappa, Clare Finney, Sinead Heyler, Emma Clarey, Maeve Cockrell, Maria Therese Depante, Kevin O’Reilly, Christopher Waterhouse, Vicky Chu, Joanne Gordon, Greg Marchant, Stuart Chandler, Senait Haile, Joanna Flanagan, Gaynor Notcheva, Dom Jimenez, Dilbagh Gill, Sunil Jamadarkhana, Sunita Gohil, Sura Dabbagh, Katy-Jane Chick, Carina Cruz, Vikram Anumakonda, Vanessa Moore, Lisa Stanton, Sharon Westwood, Jacqueline Smith, Karen Reid, Ranjit Gidda, Elena Anastasescu, Karim Salem, Mohamed Mooradun, Michael Reay, Nasirul Ekbal, Margaret McNeil, Helder Filipe, Aarti Nandani, Glykeria Pakou, Mark Neef, Sara Mingo, Amitaa Maharajh, Prashanth Nandhabalan, Thomas Billyard, Laura Wild, Pamela Bremmer, Geraldine Ward, Fiona McGurk, Rajbinder Deol, Catherine Morgan, Kirandeep Pachoo, Barbara Phillips, Owen Boyd, Claire Phillips, Rakhee Hindocha, Stephen Drage, John Porter, Alex Harrison, Lynn Evans, Lousie Ortiz-Ruiz de Gordoa, Dominika Wlazly, Tomas Andrews, Jess West, Ben Attwood, Paul Jefferson, Penny Parsons, Sophie Mason, Bridget Campbell, Julia Jones, Kathryn Webb, Karen Spicer, Angela Day, Camilla Stagg, Noor Ayesha Shah, Ian Purcell, Lucy Shafiq, Coralie Carle, Rebecca Chilvers, Heather MacColl, Alan Pope, Andrew Holder, Nicola Butterworth-Cowin, Matthew Davies, Louise Wilmer, Nadeem Ismail, Sneha Gurung, Piers Murphy, Toby Elkington, Matthew Camilleri, Rob Charnock, Claire Shevlin, Emma McGregor, Chris Clarke, Judith Hinds, Sophie Hughes, Raymond McKee, Denise McFarland, Roisin McNulty, Andy Breen, Elizabeth Wilby, Nora Youngs, Richard French, Suzie Colquhoun, Claire Posnett, Clare Howcroft, Andrew Taylor, Simon Whiteley, Bethan Ogg, Kate Long, Alicia Tomkinson, Ian Clement, Tara Shrestha, Leigh Dunn, Verity Calder, Maite Babio-Galan, Kimberley Zwiggelaar, Anne Mitchell, Julie Stephenson, Lesley Rigden, Jude Davison, Joseph Carter, Kate Howard, Hazel Cahill, Lia Grainger, Poppy Cottrell-Howe, Abigail Rowbotham, Laura Jeffery, Emily Waterman, Arran Fletcher, Zoe Guy, Isabel Birkinshaw, Jo Ingham, Zoe Scott, Samantha Stead, Raha West, Pradeep Shanmugasundaram, Judith Abrams, Geraldine Hambrook, Katarina Manso, Sally Scott, Iram Husain, Evelyn Chan, Siobhan Gettings, Anil Hormis, Rachel Walker, Dawn Collier, Cheryl Graham, Victoria Murray, Katy Curtis, Charlotte Widdop, Sarah Kimpton, Susan Oakley, Zirlish Afzal, James Varley, Petra Polgarova, Andrew Johnston, Lynne Whitehead, Andrew Conway Morris, Razeen Mahroof, Sofia Teixeira, Nazrudeen Ali, Jonny Wilkinson, Alex Lyon, Charlotte Mills, Kathryn Hall, Gayna Grantham, Lorraine Campey, Helen Cronshaw, Livia Malanjum, Lucy Dudgeon, Clare Hinchley, Stephen Langhon, Jane Hosea, Andrea Hillyer, Rachel Kontogonis, Oninye Ndefo, Laura Robinson, Callum Kaye, Kevin Sim, Ian Scott Teresa Scott, Felicity Anderson, Wendy Mitchell, Patricia Cooper, James MacBrayne, Fiona Willox, Kate Richmond, Rebecca Colleron, Erin Trumper, Bartosz Was, Michael Christie, Amber Johnson, Gillian Price, Malcolm Sim, Robert Docking, Scott McQueen, Sophie Kennedy-Kay, Lynn Abel, Sophie Hughes, Steven Henderson, Kirsty McLeash, Andrew Gratrix, Victoria Martinson, Louise Foster, Elizabeth Stones, Vicki Lowthorpe, Daniel Crawley, Susannah Leaver, Sarah Farnell Ward, Romina Pepermans Saluzzio, Frances Robinson, Carlos Delgado, Geraldine Gray, Rebecca Kanu, Robert Oakley

**Affiliations:** 1 Institute of Inflammation and Ageing, College of Medical and Dental Sciences, University of Birmingham, Birmingham, UK; 2 Department of Biochemical Engineering, School of Chemical Engineering, College of Engineering and Physical Sciences, University of Birmingham, Birmingham, UK; 3 Warwick Clinical Trials Unit, University of Warwick, Coventry, UK; 4 University Hospitals Birmingham NHS Foundation Trust, Birmingham, UK; 5 Division of Anaesthetics, Pain Medicine and Intensive Care, Faculty of Medicine, Imperial College London, London, UK; 6 Kadoorie Centre for Critical Care Research, Nuffield Division of Anaesthesia, University of Oxford, Oxford, UK; 7 Regional Intensive Care Unit, Royal Victoria Hospital, Belfast Health and Social Care Trust, Belfast, UK; 8 The Wellcome Wolfson Institute for Experimental Medicine, Queens University Belfast, Belfast, UK; 9 Department of Medicine and Wolfson Institute for Biomedical Research, Centre for Intensive Care Medicine, University College London, London, UK; 10 Department of Life Sciences, Aberystwyth University, Ceredigion, UK

**Keywords:** STRESS-L, β-Blocker, Sepsis, Septic shock, Metabolomics, Cytokines, Clinical trial

## Abstract

**Purpose:**

The landiolol and organ failure in patients with septic shock (STRESS-L study) included a pre-planned sub-study to assess the effect of landiolol treatment on inflammatory and metabolomic markers.

**Methods:**

Samples collected from 91 patients randomised to STRESS-L were profiled for immune and metabolomic markers. A panel of pro- and anti-inflammatory cytokines were measured through commercially acquired multiplex Luminex assays and statistically analysed by individual and cluster-level analysis (patient). Metabolite fingerprinting was carried out by flow infusion electrospray ionisation high-resolution mass spectrometry and metabolomic data were analysed using the R-based platform MetaboAnalyst. The metabolites were identified using DIMEdb (dimedb.ibers.aber.ac.uk) from their mass/charge ratios. These metabolomic data were also re-analysed using individual and cluster-level analysis. The individual-level models were adjusted for confounders, such as age, sex, noradrenaline dosage and patient (random effect).

**Results:**

Analysis was undertaken at cluster- and individual-level. There were no significant differences in cytokine concentration level between trial arms nor survivors and non-survivors over the duration of the observations from day 1 to day 4. Metabolomic analysis showed some separation in the levels of ceramides and cardiolipins between those who survived and those who died. Following adjusted analysis for confounders, plasma metabolite concentrations remained statistically different between landiolol and standard care arms for succinic acid, l-tryptophan, l-alanine, 2,2,2-trichloroethanol, lactic acid and d-glucose.

**Conclusions:**

In a study of ICU patients with established septic shock and a tachycardia, landiolol treatment used to reduce the heart rate from above 95 to a range between 80 and 94 beats per minute did not induce significant cytokine changes. d-Glucose, lactic acid, succinic acid, l-alanine, l-tryptophan and trichloroethanol were pathways that may merit further investigation.

*Trial Registration*: EU Clinical Trials Register Eudra CT: 2017-001785-14 (https://www.clinicaltrialsregister.eu/ctr-search/trial/2017-001785-14/GB); ISRCTN registry Identifier: ISRCTN12600919 (https://www.isrctn.com/ISRCTN12600919).

**Supplementary Information:**

The online version contains supplementary material available at 10.1186/s40635-024-00708-6.

## Introduction

The search for interventions that can modulate the dysregulated response in sepsis have been underway for more than 30 years. The report of a greatly reduced mortality with the use of esmolol [1] raised the possibility that beta-blockade could modulate the immune system during septic shock.

Although the mainstay of blood pressure management in patients with septic shock is noradrenaline, there is a growing narrative suggesting harm from its administration and sympathetic activation during septic shock has been reported in both animal models [2–6] and patients [7, 8]. However, the recent STRESS-L study [9], which compared the addition of landiolol (AOP Health, Vienna, Austria) to standard care in patients with established septic shock, found no difference in organ support in the 14 days following randomisation. STRESS-L was terminated at 37% of full recruitment because of concerns of harm which included increased lactate, increased noradrenaline use and increased mortality in the landiolol group.

Landiolol was selected as the intervention as it is highly specific for the β_1_-receptor—some 200 times greater compared with esmolol [10, 11]. Changes in beta-receptor density with catecholamine stimulation [12, 13] mean that relative effects of β1- or β2-blockade is not predictable. Whilst short-term (< 24 h) animal models have suggested that the immune response may be attenuated by β2-blockade (reviewed [14]), this may not be the case once sepsis has become established for longer than 24 h. Immune modulation by beta-blockade has been reported in septic rats in a 5-day model using the antagonism of the β1-receptor with metoprolol [5] reduced pro-inflammatory cytokines and prolonged survival times. Whilst esmolol improved survival at 120 h in a mouse model of sepsis [15] which the authors explained by activation of immune response and cell repair pathways through NFKB and BRCA1 genes. Landiolol has been shown to decreased circulating cytokines, TNF-alpha, IL-6, and high mobility group box (HMGB)−1 and reduced histological lung damage in a rat endotoxin model [6]. Furthermore, it was shown to be cardio-protective in septic rats by normalising the expression of cardiac vasoactive peptide endothelin-1 [16].

STRESS-L recruited patients who had been exposed to high doses of noradrenaline for at least 24 h and were tachycardic and planned [17] to perform exploratory investigations to characterise differences in plasma cytokine concentrations and metabolomics associated with landiolol treatment. To our knowledge, there have been no randomised studies in humans reporting these changes associated with septic shock and treatment with beta-blockade.

## Methods

The protocol for STRESS-L has been previously published [17] and was approved by the East of England, Essex Research Ethics Committee (Reference, 17/EE/0368). Patients with septic shock, treated with noradrenaline for more than 24 h and, at randomisation, a tachycardia of 95 beats per minute (bpm) or more, and treated with a noradrenaline dose of 0.1 mcg/kg/min or more, were randomised on a 1:1 ratio to receive standard care or standard care plus a landiolol infusion between 1 and 40 mcg/kg/min to control the heart rate between 80 and 94 bpm.

### Patient cohorts and interventions

Blood samples were collected on days 0, 1, 2, 4 and end of noradrenaline therapy (EoNT). Day 0 samples were obtained pre-infusion of landiolol in those randomised to the interventional arm. EoNT was defined as the timepoint 12 h after the noradrenaline infusion was stopped. Once this point had been reached, no further bloods were drawn even if EoNT was before Day 4 or Day 6. Sampling stopped once the patient had died or been discharged from ICU.

At study sites, whole blood vacutainers containing EDTA were centrifuged at room temperature for 10 min at 1500×*g*. The plasma layer was removed and transferred to cryovials in 500 μL aliquots. Plasma samples were then initially stored at – 20 ⁰C (for example where the sample was taken on a weekend) and moved as soon as possible to long-term storage at − 80 ⁰C. Samples were batch transferred to central laboratories for analysis where the analyst was blinded to the treatment allocation.

Although all samples were analysed, only results from the first 96 h following randomisation are reported as the sample numbers beyond this were small and patients who remained on noradrenaline were often being treated for secondary nosocomial infection and had secondary changes in cytokines.

### Cytokine measurements

Plasma cytokine concentrations (interferon-gamma (IFNγ), interleukin (IL)−2, IL-4, IL-5, IL-6, IL-8, IL-10, IL-12 p70, IL-17, interleukin-1 receptor antagonist (IL-1Ra) and tumour necrosis factor-alpha (TNF-α) cytokines) were measured using a commercial Luminex Discovery Assay kit according to manufacturer’s instructions (#LXSAHM-12; Bio-techne, UK). Analysis was completed using Bio-Plex Manager (v2.1).

### Metabolite fingerprinting by flow infusion electrospray ionisation high-resolution mass spectrometry (FIE-HRMS)

Plasma samples in 2-mL microcentrifuge tubes were centrifuged at 22,000×*g* at 4 °C for 10 min. Then 100 µL of the supernatant was transferred into mass spectrometry vials along with 100 µL methanol/water [70/30 *v/v*]. For each sample, 20 µL was injected into 70% water/ 30% methanol at a flow of 60 µL per minute, using a Surveyor flow system into a Q Exactive plus mass analyser instrument with a UHPLC system (Thermo Fisher Scientific^©^, Bremen, Germany). Data acquisition for each sample was in alternating the positive and negative ionisation modes, throughout four mass/charge ratios (*m/z*) ranges (15–110 *m/z*, 100–220 *m/z*, 210–510 *m/z*, 500–1200 *m/z*) with an acquisition time of 2 min. The peaks data were filtered based on relative standard deviation (RSD) of 0.5 to derive *m/z* bins. The *m/z* data were further normalised based on total ion count (TIC) using the R package metabolyseR v0.14.10 (Finch, 2022 metabolyseR: Methods for Pre-Treatment, Data Mining and Correlation Analyses of Metabolomics Data. https://github.com/jasenfinch/metabolyseR).

Metabolomic data were analysed using R-based web platform MetaboAnalyst 4. Data were subjected to interquartile range-based filtering, log_10_ transformations and Pareto scaling. Chemometric analyses (principal component analysis (PCA), partial least squares-discriminant analyses (PLS-DA)), ANOVA and variables of importance for the projection (VIP) scores (> 1) were conducted. Major sources of variation were displayed using unsupervised hierarchical clustering analysis (HCA). Area under the curve (AUC) assessments based on sensitivity and specificity estimates were used to suggest the accuracy of the targeted *m/z* as potential markers of pathways through which landiolol may be exerting an effect. Targeted *m/z* were related to discrete metabolites based on accurate mass (± 5 ppm) using the DIMEdb database (https://dimedb.ibers.aber.ac.uk/) considering their ionised masses, molecular formulae. All isotopes/adducts were considered in deriving the identifications. Correlation analyses between identified metabolites and cytokines were based on Pearson’s coefficients.

### Statistical analysis of cytokine data

Skewed data underwent logarithmic conversion prior to analysis and were reconverted for reporting. Following analysis, samples which were below the limit of detection were not included in further analyses. The data were analysed using both cluster-level analysis and individual-level analysis methods. In cluster-level analysis, the mean of each cytokine variable for each participant over the timepoints (days 0, 1, 2, 4) was calculated and then the mean of means for each variable was compared between the arms using a standard t-test. In individual-level analysis, linear mixed-effects models were fitted for each variable to estimate the treatment difference, 95% CI, and *p*-value. The models were adjusted for age, sex and baseline noradrenaline, and patient ID (random effect).

Where cytokine data were analysed integrated with metabolomic data, no specific data modelling was applied (i.e., models were not adjusted for age, sex, baseline noradrenaline levels, and with no random effect), and was processed through MetaboAnalyst. Data were subjected to interquartile range-based filtering, log_10_ transformations and Pareto scaling.

## Results

Samples were obtained from 91 patients (43 treated with landiolol and 48 standard care). The mean age was 55.31 ± 16.7 [years, mean ± SD] (55.1 ± 14.9 landiolol; 55.4 ± 18.4 standard care). 55 of the 91 patients (60%) were men (27 (63%) landiolol; 28 (58%) standard care). The median noradrenaline dose at randomisation was 0.27 (0.19–0.45) [mcg/kg/min, median (interquartile range, IQR)] (0.25 (0.17–0.44) landiolol; 0.30 (0.21–0.45) standard care. The mean heart rate at randomisation was 110.3 ± 11.7 [bpm, mean ± SD] (109.5 ± 10.5 landiolol arm; 111.0 ± 12.7 standard arm).

60 patients (65%) were treated with steroids (27 (63%) landiolol; 33 (69%) standard care). 13 patients received additional beta-agonists (adrenaline or dobutamine) (8 (19%) landiolol; 5 (10%). Table 1 summarises the values of cytokine concentrations at randomisation. There were no statistical differences between study arms.

**Table 1:** The treatment effect (95% CI) and *p*-values using cluster-level and individual-level analyses

Variable	Cluster-level analysis				Individual-level analysis				
	Standard arm	Landiolol arm	*p*-value	*p*-value	Adjusted analysis				
	N = 48	N = 43	(t-test)	(non-para)					
	Mean (SD)	Mean (SD)			Estimate	SE	LL	UL	p-value
							(95% Cl)	(95% Cl)	
IL-2	25.2 (85.8)	8.6 (15.1)	0.215	0.701	− 15.7	13.4	− 42	10.6	0.241
IL-8	284.3 (688.2)	319.7 (687.7)	0.807	0.949	27.1	129.9	− 227.6	281.7	0.835
IL-12	281.2 (987.8)	73.3 (150)	0.176	0.59	− 195.5	154.8	− 498.9	107.8	0.206
TNF-α	28.7 (78.5)	22.9 (54.4)	0.685	0.352	− 6.1	14.2	− 33.9	21.6	0.665
IL-1Ra	4186.7 (5460.5)	65,754.1 (383,963.4)	0.269	0.049	52,387.2	45,943.8	− 37,661	142,435	0.254
IL-4	39.2 (51.3)	34.5 (36.5)	0.615	0.849	− 4.7	9.4	− 23.1	13.7	0.617
IL-6	676.1 (1803)	2056.3 (9373.5)	0.32	0.668	1006	1009.3	− 972.1	2984.1	0.319
IL-10	26 (46.7)	88.7 (273.1)	0.121	0.633	58.5	43.8	− 27.4	144.4	0.182
IL-17	12.8 (27.8)	9.0 (28)	0.51	0.731	− 4.3	5.3	− 14.6	6	0.411

### Cytokine assessment of landiolol on the STRESS-L population

**Fig. 1:**
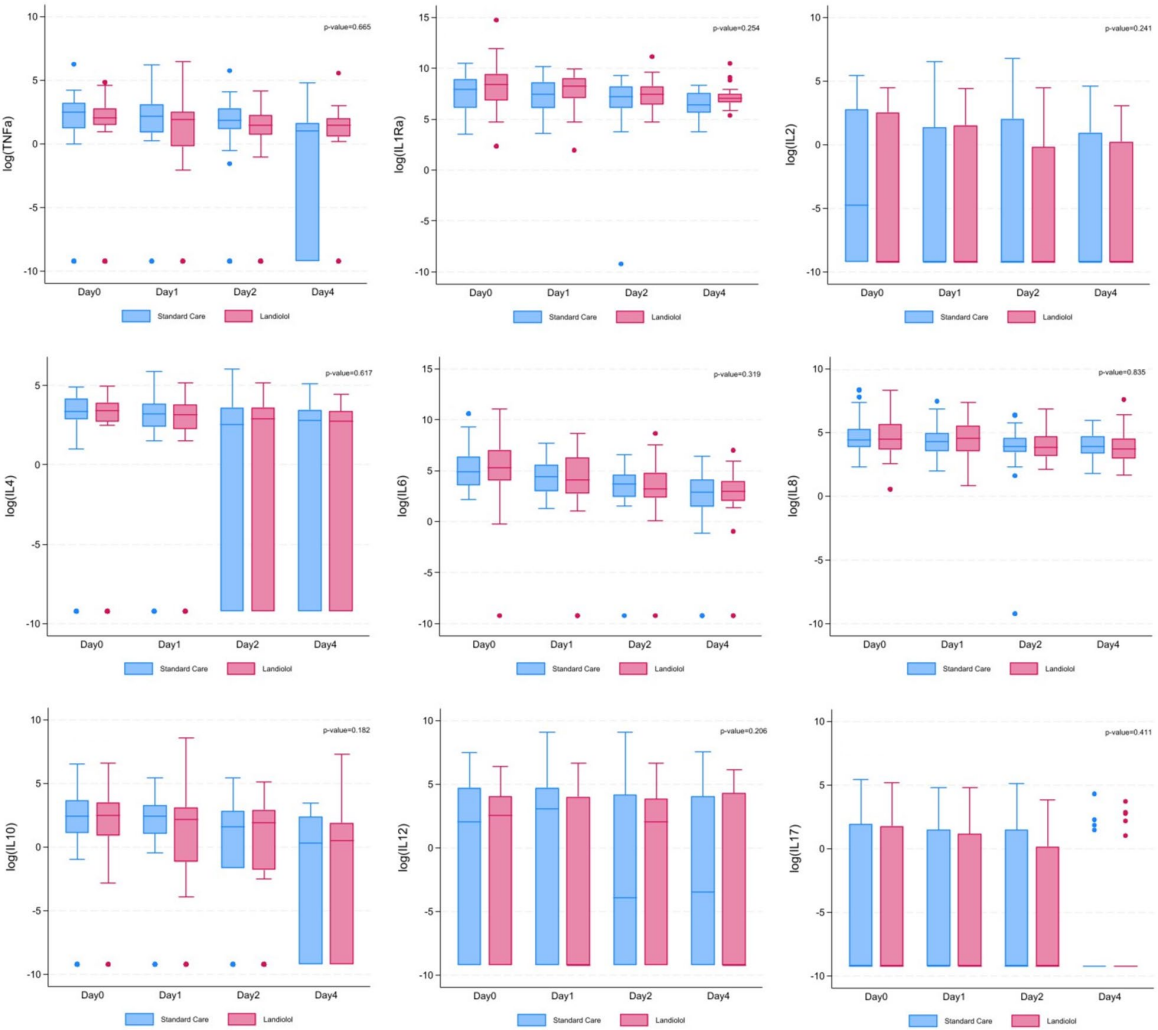
Box and whisker plots of cytokines concentrations between patients assigned by treatment. All cytokine concentrations expressed as pg mL^−1^. The *p*-values inserted into the plots are from the individual-level adjusted analysis to test if the treatment effects are significantly different from zero

The changes in cytokine concentrations over time and between groups are summarised in Table 1 and Fig. 1. There were no significant differences between groups for any cytokine with either cluster-level or individual-level analysis with time. Similarly, there were no significant differences between groups and those who went on to survive compared with those who died.

### Metabolomic assessment of landiolol on the STRESS-L population

**Fig. 2:**
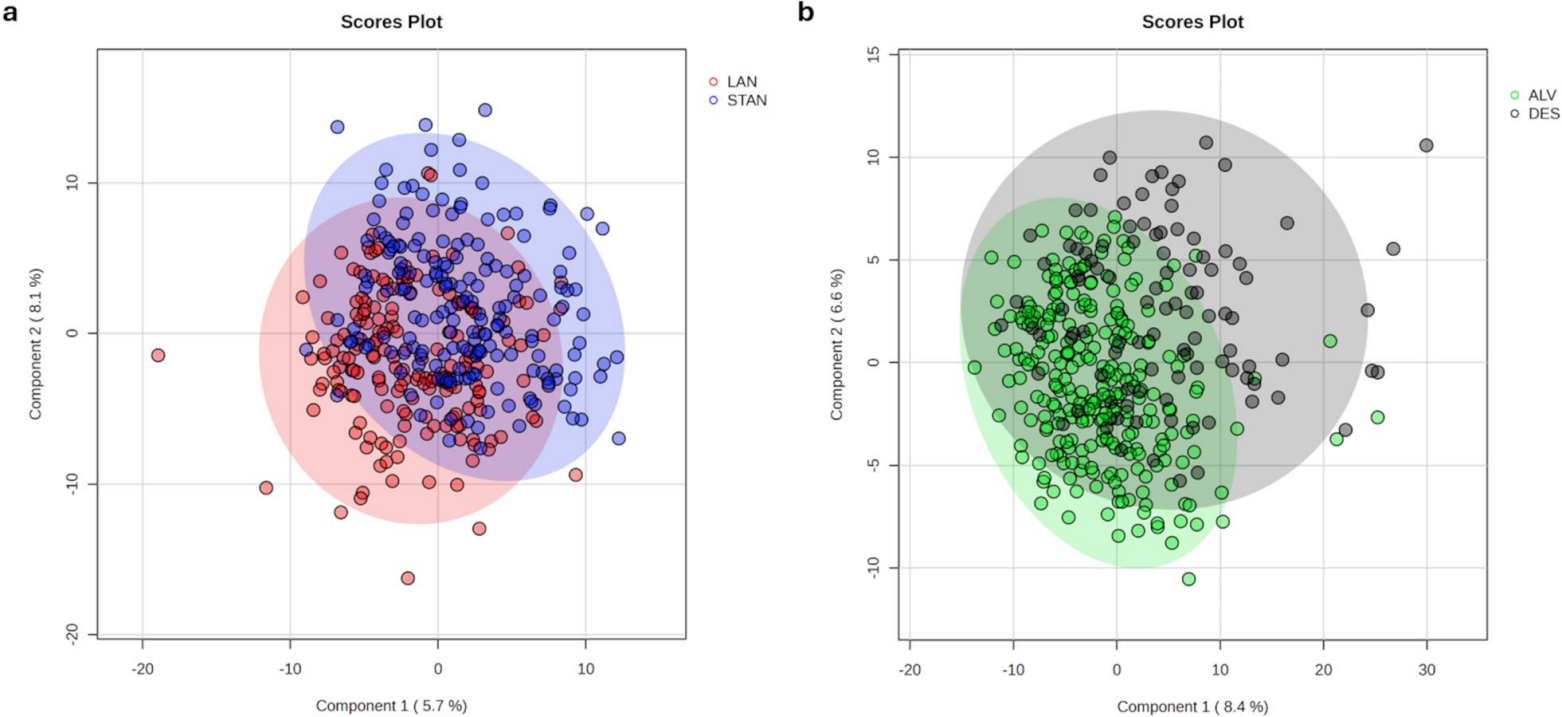
Partial least square discriminant analysis (PLS-DA) of plasma metabolomes from patients assigned by treatment allocation (**A**) or 28-day patient mortality data (**B**). *LAN* landiolol, *STAN* standard care, *ALV* alive, *DES* deceased

PLS-DA assessments considered the data based on treatment allocation (Fig. 2a) and 28-day patient mortality data (Fig. 2b). Separation of landiolol (LAN) from standard (STAN) group led to the derived model which was tested using leave-one-out cross-validation (LOOVC) to indicate the derived PLS-DA has predictive power (*Q*^2^ = 0.51). Considering the data stratification based on mortality data, the samples from the DES class showed some evidence of clustering away from the ALV class (*Q*^2^ = 0.47) (Fig. 2b) suggesting that samples had detectable differences even if there were few differences between LAN and STAN.

The major sources of variation PLS-DA analysis by study arm were assessed and significant differences were identified in the LAN versus STAN comparison (Supplementary Table). These suggested changes in ceramide (Cer), cardiolipid (CL) and phosphatidylethanolamine (PE) lipid classes. Groups were also compared by mortality (ALV—alive; DES—dead), more significant differences were observed if all four classifications (LAN, STAN, ALV, DES) were considered together and the major sources of variation identified (Table 2).

**Table 2:** Significant changes in metabolites distinguishing patients assigned by treatment allocation or 28-day patient mortality data

Name	*f*.value	*p*.value	− LOG10(*p*)	FDR
**Succinic acid**	**9.966**	** < 0.001**	**5.600**	** < 0.001**
Ketoleucine	9.584	< 0.001	5.377	< 0.001
**l-Tryptophan**	**8.596**	** < 0.001**	**4.799**	** < 0.001**
**l-Alanine**	**8.585**	** < 0.001**	**4.793**	** < 0.001**
**2,2,2-Trichloroethanol**	**8.190**	** < 0.001**	**4.561**	** < 0.001**
All-trans-decaprenyl diphosphate	8.114	< 0.001	4.517	< 0.001
**Lactic acid**	**7.593**	** < 0.001**	**4.211**	** < 0.001**
Estrone sulfate	7.500	< 0.001	4.156	< 0.001
Cortisol	6.702	< 0.001	3.686	< 0.001
Normetanephrine	6.614	< 0.001	3.634	< 0.001
**d-Glucose**	**6.612**	** < 0.001**	**3.633**	** < 0.001**
Tetrahydrocortisone	6.027	< 0.001	3.288	0.001
*N*-Acetyl-d-glucosamine	5.908	< 0.001	3.218	0.001
l-Tyrosine	5.310	0.001	2.865	0.003
Alpha-ketoisovaleric acid	5.256	0.001	2.834	0.003
Dihomo-gamma-linolenic acid	5.131	0.002	2.760	0.003
Stearidonoyl CoA	5.094	0.002	2.738	0.003
CMP-2-aminoethylphosphonate	5.001	0.002	2.684	0.004
Phenylacetic acid	4.890	0.002	2.618	0.004
Pyruvic acid	4.873	0.002	2.608	0.004
Stearic acid	4.762	0.003	2.543	0.004
Sucrose	4.626	0.003	2.463	0.005
Cholesterol sulfate	4.591	0.004	2.442	0.005
Dehydroepiandrosterone sulfate	4.469	0.004	2.370	0.006
Glycocholic acid	4.344	0.005	2.297	0.006
Formic acid	4.203	0.006	2.215	0.007
Didemethylcitalopram	3.966	0.008	2.076	0.010
Arachidonic acid	3.896	0.009	2.035	0.010
3-Hydroxybutyric acid	3.843	0.010	2.004	0.011
Caprylic acid	3.714	0.012	1.929	0.012
Prostaglandin E2	3.587	0.014	1.854	0.014

Those highlighted in bold remained significant following either individual-level or cluster-level analysis. Post hoc analysis for the metabolites can be found in the supplementary information

**Fig. 3:**
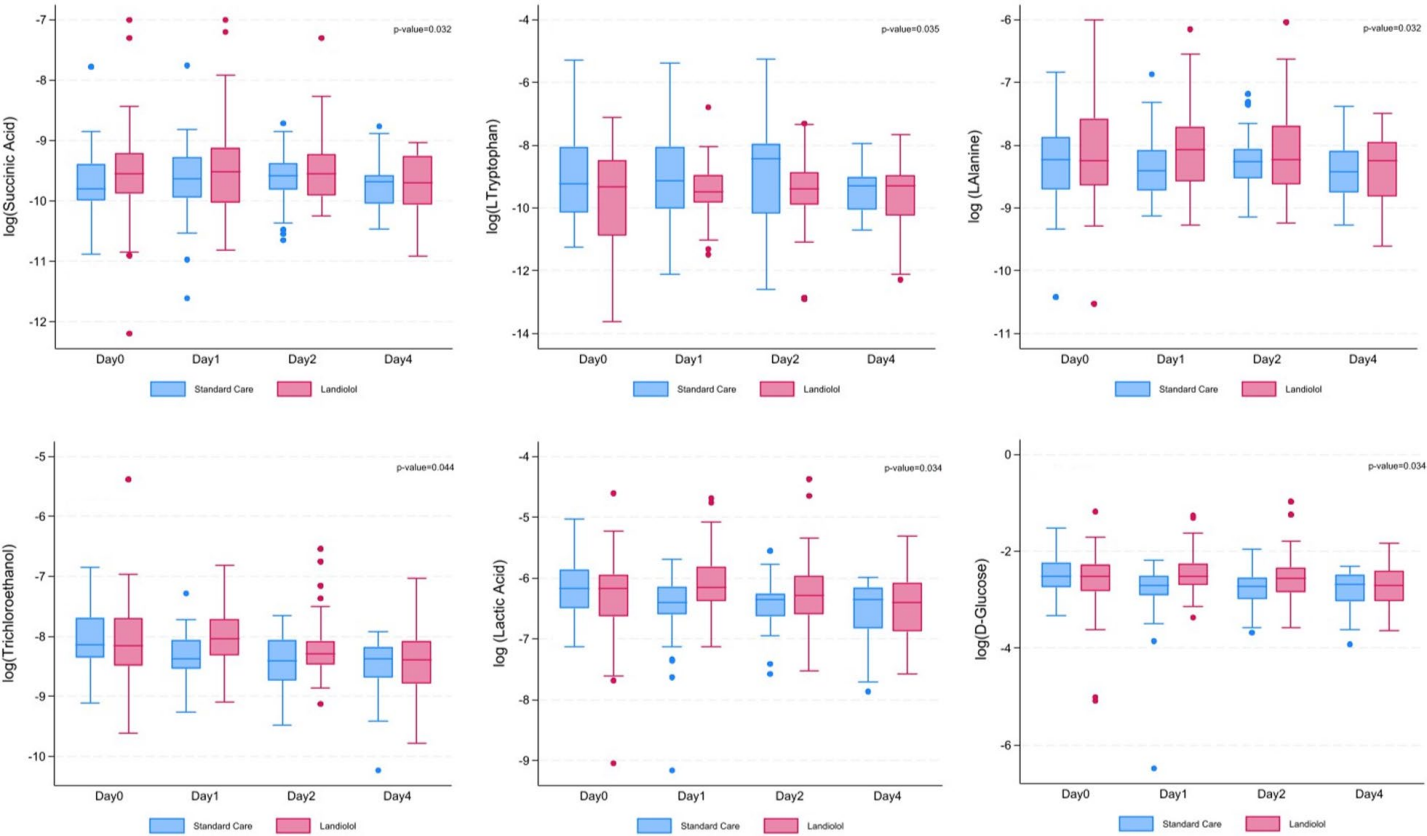
Box and whisker plots of statistically significant metabolites as denoted from individual and cluster-level analysis. The *p*-values inserted into the plots are from the individual-level adjusted analysis to test if the treatment effects are significantly different from zero

The metabolomic analysis was initially performed without taking into consideration baseline metabolite concentrations, sex, noradrenaline dosage and site effect, and as observed during the cytokine analysis, statistical significance was reached in our dataset at global levels and often between groups, but not necessarily at individual timepoints. Here also, further cluster-level analysis and individual-level analysis were applied using a mixed-effect model. Adjusted for the same conditions as the cytokine analysis, 5 metabolites were significantly different between the trial arms by *t*-test: d-glucose (*p* = 0.040), lactic acid (*p* = 0.040), l-alanine (*p* = 0.037), l-tryptophan (*p* = 0.045) and succinic acid (*p* = 0.023). For adjusted analysis, here 6 significant metabolites were noted, 5 remaining from the previous analysis (d-glucose [*p* = 0.034], lactic acid [*p* = 0.034], succinic acid [*p* = 0.032], l-alanine [*p* = 0.032] and L-tryptophan [*p* = 0.035]) and a new significant metabolite, trichloroethanol (*p* = 0.044). Over representation analysis was conducted on these 6 metabolites, which still demonstrate glucose-alanine pathway and Warburg effect enrichment. Results also showed a trend towards significance for CMP-2-aminoethylphosphonate (*p* = 0.053) and onward trend for ketoleucine (*p* = 0.075). Statistically significant metabolites are presented in Fig. 3 and Table 2.

## Discussion

STRESS-L was a study of ICU patients with established septic shock and a tachycardia, landiolol treatment used to reduce the heart rate from above 95 to a range between 80 and 94 beats per minute did not induce significant changes in cytokine or metabolite levels. Although we report separation of markers between patients who died compared with those who survived, we found no systematic separation by treatment allocation. It would appear that there were no systemic cytokine pathways that were changed by beta1-blockade with landiolol and so we conclude that the findings of STRESS-L were not due to generalised immunomodulation.

The variability of cytokine response between patients is high. A recent analysis of samples and clinical data from two septic shock studies (LeoPARDS [18] and VANISH [19]) and the UK Genomic Advances in Sepsis (GAinS) [20], reported clustering related to severity, but not treatment response [21]. Recent identification of molecular phenotypes [22] using latent class analysis may offer an alternative way to identify treatment effects with these data, as may further stratification of the cohort assessing severity by cytokine level rather than noradrenaline dose.

As samples were collected and frozen for central analysis, it was not possible to measure the effect of beta-blockade on immune cellular function. Signalling through beta2-receptors suppresses pro-inflammatory cytokine secretion from both macrophages and dendritic cells in response to lipopolysaccharide [23, 24]. IL-10 may be induced [25, 26] and acts as an autocrine hormone to block TNF-alpha and other inflammatory cytokines [26]. Whilst pre-clinical data suggested that antagonism of beta1 pathways may preferentially expose more beta2 effects, this was not demonstrated by this study or the clinical study [9]. A better understanding of the changes in differential beta-receptor densities and the size of the effect when these receptors are agonised by noradrenaline in the clinical setting, is needed.

Metabolic pathways were affected by beta1-blockade but more patients were treated with adrenaline or dobutamine in the landiolol group and these treatments could account for increases in lactate, pyruvate and Warburg effect metabolites. STRESS-L was stopped in part because of increased lactate concentrations and this was confirmed on metabolomic screening. The increased D-glucose concentrations are a known side effect of beta-blockade.

Changes in ceramide (Cer), cardiolipid (CL) and phosphatidylethanolamine (PE) lipid classes may reflect mitochondrial dysfunction. Energetic failure through mitochondrial dysfunction has been proposed as a driver of organ failure in sepsis [27]. Cer and CL have been found to regulate autophagy [28] and differences between landiolol treated patients and standard care as well as between those who died and survived may reflect possible areas of future research. Furthermore changes in L-alanine, L-tryptophan and succinic acid may merit further investigation, however it is also likely that, given the high number of comparisons and the lack of changes in co-existing pathways, these results may have occurred by chance.

This study only compared cytokines and metabolites in plasma at the time of infusion and over the 4 days following randomisation. It would appear that landiolol did not, on the number of patients studied, influence any immediate cytokine or metabolic changes. This calls into question whether there is any short-term mechanism through which landiolol treatment of patients treated with noradrenaline and a tachycardia may act. Furthermore, it is unknown whether there are intra-cellular changes that have been set in motion but undetectable in plasma. This study does not address whether there may be longer-term effects that have been unmeasured.

### Limitations

There were several limitations to our study. First, our findings could have been different had the landiolol administration been started at a different timepoint or at a different dose of noradrenaline. Second, it is not possible to infer whether these findings are a class effect, applicable to all beta-blocking drugs or due to the high specificity for the β1-receptor of landiolol. Third, by stopping prematurely for suspected harm, the trial may not have sufficient power to describe clinically significant pathways. Fourth, the mortality was measured at 28 days and it may be that the cytokines and metabolites measured on the first four days following randomisation were too far removed from the 28-day outcome to offer meaningful interpretation. Fifth, this paper does not investigate possible intra-cellular mechanisms not detected in plasma.

## Conclusions

In a study of ICU patients with established septic shock and a tachycardia, landiolol treatment used to reduce the heart rate from above 95 to a range between 80 and 94 beats per minute did not induce significant cytokine or metabolomic changes. As STRESS-L was terminated before its full recruitment, the study may be underpowered to identify significant pathway differences.

## Supplementary Information

Supplementary Material 1

## Data Availability

Data are available on application to the chief investigators via email: Prof Tony Whitehouse (Tony.Whitehouse@uhb.nhs.uk).
