# Supplementary material for: The effects of ultra-selective beta1-antagonism on the metabolic and cytokine profile in septic shock patients receiving noradrenaline: a sub-investigation from the STRESS-L Randomised Study
Source: Intensive Care Med Exp. 2025 Jan 22;13:9. doi: 10.1186/s40635-024-00708-6 (PMC11754546; doi:10.1186/s40635-024-00708-6)
Supplement: Supplementary file 1 — Supplementary Material 1 [file 40635_2024_708_MOESM1_ESM.docx]

Supplementary Table: Adjusted analysis for statistically significant (or trending towards significance (§)) for treatment arms, * based on initial unadjusted analysis, (+) greater in landiolol (-) lower in landiolol treated patients.

| Metabolite | Statistical Test | Adjusted result | Change relative to landiolol* |
| --- | --- | --- | --- |
| *m/z* 243.10382 | Estimate (se) | -0.17 (0.06) | - |
|  | p-value | 0.003 |  |
|  | 95% Cl | (-0.30, -0.06) |  |
| *m/z* 269.71368^§^ | Estimate (se) | 0.09 (0.05) | + |
|  | p-value | 0.06 |  |
|  | 95% Cl | (-0.003, 0.19) |  |
| Cer (d18:1/16:0) | Estimate (se) | -0.08 (0.04) | - |
|  | p-value | 0.05 |  |
|  | 95% Cl | (-0.16, 0.0003) |  |
| Cer(d18:0/26:0) | Estimate (se) | 0.45 (0.11) | + |
|  | p-value | <0.0001 |  |
|  | 95% Cl | (0.24, 0.66) |  |
| CL(i-12:0/i-22:0/i-15:0/18:2(9Z,11Z)) | Estimate (se) | -0.07 (0.04) | - |
|  | p-value | 0.05 |  |
|  | 95% Cl | (-0.14, 0.001) |  |
| CL(i-13:0/i-24:0/18:2(9Z,11Z)/i-24:0) | Estimate (se) | 0.13 (0.05) | + |
|  | p-value | 0.004 |  |
|  | 95% Cl | (0.04, 0.22) |  |
| PE(14:0/P-18:0) | Estimate (se) | -0.03 (0.01) | - |
|  | p-value | 0.05 |  |
|  | 95% Cl | (-0.05, 0.0001) |  |
| Ribose-1,5-bisphosphate^§^ | Estimate (se) | -0.08 (0.05) | - |
|  | p-value | 0.08 |  |
|  | 95% Cl | (0-18, 0.008) |  |
